# Dine Early and Eat Less

**Published:** 1875-05

**Authors:** Uncle Frank


### DINE EARLIER AND EAT LESS.

This was Abernethy's advice to a woman who consulted him about her stomach. She was a heavy eater, and was especially fond of night suppers, comprising many dishes, all so highly seasoned as to secure a new sensation to the palate, even after the appetite for food had been satisfied. Then, to increase the desire and the ability to swallow food, she drank wine and other stimulating beverages, and was thus able to crowd down a tremendous mass of miscellaneous material. Of course the stomach tried to take care of the load, in the ordinary way. It strove to digest it. It put forth all its powers and drew upon its best gastric resources. It demanded from the blood more gastric juice with which to dissolve the inordinate mass. It labored long and earnestly, but without avail. Its plea for help adequate to the great burden imposed, could not be met. Natural digestion was impossible, because the mass was not only too great, but because it was not chiefly composed of digestible material.

How were the contents of that misused stomach disposed of? They were parcelled out and got rid of, after a fashion. The alcohol in the wine was absorbed at once into the circulation and was presently evolved from the lungs. Of course it was a great comfort to get rid of this irritant, and if no other irritant had been present, and the stomach had been less completely gorged, digestion could speedily have been accomplished. But under the circumstances, about the only thing to be done, was to allow the mixture to ferment, and, after occasioning from twelve to forty hours of misery, to be evacuated in an undigested form, leaving the stomach and alimentary canal all the worse for the transit.

Abernethy's injunction to dine earlier and eat less, was a sound one ; but, as will be seen, it did not touch the whole evil. Abernethy was a wise man in many respects ; but he is said to have got drunk now and then, in spite of the severity of his strictures upon those of his patients who indulged in excessive eating and drinking. He was an advanced thinker, and had he lived to-day, he would probably have added to his "Dine Earlier and Eat Less" direction, these words : "And see to it that you avoid wines and spices and all stimulants."

It is very difficult for the average human being to eat moderately, if many kinds of food are eaten. Each has its own peculiar flavor, and the palate is tickled and gratified by being invited to partake of one after another, even though the stomach has been meantime sufficiently burdened. If some biting, heating substance is swallowed, such as wine or brandy or spice or pepper, a sensation is imparted which is mistaken for appetite, or craving for food, and the person appears to feel hungry. But this sensation is wholly artificial, and cannot be safely acted upon. This heat and false hunger can be kept up by stimulants until the stomach is distended to its utmost limit. It is, in fact, often kept up until vomiting supervenes, and in such cases relief comes with little delay. If vomiting does not follow over-eating—as it does not once in a hundred times—the slow and tormenting fermentive process is about the only recourse left, and every dyspeptic knows how wretched this process can render a human being.

Perhaps the silliest thing in life is to expect to give the overworked stomach strength through medicine. It would be altogether funny if it were not so serious an affair to hear medical men gravely suggest iron and bitters and tonics and ales, for the relief of persons who complain that their food distresses them. The truth is, that all the iron of all the mines in all the world ; all the bitters that were ever brewed from alcohol herbs and roots ; all the tonics that were ever fabricated ; all the ales which were ever made from hops and malt and muddy water—never could and never will cure a single case of dyspepsia. All these things irritate the stomach, just as they would irritate the eye if great care were not taken to keep them out of the eye. They do harm *always*, because they lessen the power of the stomach for healthy work.

The true way to be happy in the possession of a stomach is to forget that there is such an institution. Here lies the trouble. The stomach will not let you forget its existence, unless you deal reasonably with it. If you sit down to a dinner of several courses, the chances are that you will eat too much. If you barely taste of a dozen different articles of food at a meal, you probably give the stomach more to do than it is capable of doing. The temptation to eat largely is immense when the articles supplied are numerous ; and a small amount of each single article makes up a formidable aggregate.

We have heard of an old gentleman who early became a dyspeptic by the use of stimulants and over-feeding. He ran the gauntlet of "remedies," but got no permanent relief. Finally, he resorted to one meal of good, wholesome food each day, and was cured.

Not a twinge of dyspepsia for nearly twenty years!

We do not advocate one meal a day for dyspeptics. But we do say that we ordinarily expect our stomach to do more than one day's work in a day. We give it more food to digest than it can take care of, and we thus burden it oftener than is good for it. If we could let it rest at night, while we are resting, it would be a grand thing; but late dinners and suppers interfere with this. The result is, that we wake up with a general feeling of lassitude and nausea, or headache and want of appetite for the early meal. It would be otherwise if we took our heartiest meal not later than two o'clock in the afternoon and afterwards fasted till the next morning. We should find ourselves great gainers in comfort in sweet sleep, in bodily vigor, if we would eat not more than two kinds of food at each meal, not oftener than twice in twenty-four hours, and let the last meal of the day occur so long before retiring, that digestive labor need not be demanded in the hours set apart for repose. UNCLE FRANK.

